# Free Community Science and the Free Development of Science

**DOI:** 10.1371/journal.pmed.0020047

**Published:** 2005-02-22

**Authors:** Richard Stallman

**Affiliations:** Free Software FoundationBoston, MassachusettsUnited States of America

In free community science, where large numbers of scientists participate as volunteers in a single project, the ideal of scientific cooperation finds a new expression. Free community science was inspired by the free software movement, which itself was inspired by the application of the ideal of scientific cooperation, as it was applied to software development by the operating system developers of the Massachusetts Institute of Technology Artificial Intelligence Lab in the 1970s. This ideal has suffered for two decades from corporate pressure to privatize science, so it is very gratifying to see that the free software movement can today help reinvigorate the principle that inspired it.

The ideal of scientific cooperation goes beyond the conduct of individual projects. Scientific cooperation is also being reinvigorated today through the open-access movement, which promotes the public's freedom to redistribute scientific and scholarly articles. In the age of the computer networks, the best way to disseminate scientific writing is by making it freely accessible to all and letting everyone redistribute it. I give a vote of thanks to the Public Library of Science for leading the campaign that is now gaining momentum. When research funding agencies pressure journals to allow free redistribution of new articles they fund, they should apply this demand to the old articles “owned” by the same publishers—not just to papers published starting today.

Journal editors can promote scientific cooperation by adopting standards requiring internet publication of the supporting data and software for the articles they publish. The software and the data will be useful for other research. Moreover, research carried out using software cannot be checked or evaluated properly by other scientists unless they can read the source code that was used.

A significant impediment to publication and cooperation comes from university patent policies. Many universities hope to strike it rich with patents, but this is as foolish as playing the lottery, since most “technology licensing offices” don't even cover their operating costs. Like the Red Queen, these universities are running hard to stay in the same place. Society should recognize that funding university research through patents is folly, and should fund it directly, as in the past. Meanwhile, laws that encourage universities to seek patents at the expense of cooperation in research should be changed.

Another impediment comes from strings attached to corporate research funding. Universities or their public funding agencies should ensure private sponsors cannot block research they do not like. These sponsors must never have the power to veto or delay publication of results—or to intimidate the researchers. Thus, sponsors whose interests could be hurt by publication of certain possible results must never be in a position to cut the funding for a specific research group.

The free software movement, the free redistribution policy of this journal, and the practice of free community science for developing diagnostic disease classifications [1] are all based on the same fundamental principle: knowledge contributes to society when it can be shared and developed by communities. All three face opposition from those who would like to privatize knowledge and charge tolls for its use. In the free software movement we have 20 years' experience in resisting this opposition, and we have built up considerable strength and momentum. We can give the other two movements a boost, so they can advance more quickly.
